# Diagnostic accuracy of electrocardiogram algorithms for differentiating left from right outflow tract ventricular arrhythmia: a systematic review and network meta-analysis

**DOI:** 10.1136/heartjnl-2025-325916

**Published:** 2025-07-03

**Authors:** Zhuoqiao He, Ming Liu, Pengxiang Ying, Manshu Song, Xuerui Tan

**Affiliations:** 1 Department of Cardiology, First Affiliated Hospital of Shantou University Medical College, Shantou, Guangdong, China; 2 School of Medical and Health Sciences, Edith Cowan University, Joondalup, Western Australia, Australia; 3 Department of Cardiac Function, Wuhan Asia Heart Hospital, Wuhan, Hubei, China; 4 Clinical Research Center, First Affiliated Hospital of Shantou University Medical College, Shantou, Guangdong, China; 5 Human Phenome Institute of Shantou University Medical College, Guangdong Engineering Research Center of Human Phenome, Chemistry and Chemical Engineering Guangdong Laboratory, Shantou, Guangdong, China

## Abstract

**Background:**

Accurate pre-ablation differentiation between left (LVOT) and right (RVOT) ventricular outflow tract arrhythmias (OTVAs) using ECG algorithms is essential for decision on vascular access and treatment strategy. However, the most reliable ECG algorithm remains unclear. We conducted a systematic review and network meta-analysis (NMA) to compare the diagnostic accuracy of available algorithms.

**Methods:**

We searched MEDLINE, EMBASE and Cochrane databases through 7 May 2025 for studies evaluating ECG algorithms against ablation-confirmed OTVA origin. A Bayesian diagnostic test accuracy NMA was performed to estimate pooled sensitivity, specificity, diagnostic odds ratios (DORs) and a superiority index (S) for each algorithm. Study quality was assessed using the QUADAS-2 (Quality Assessment of Diagnostic Accuracy Studies) tool.

**Results:**

From 620 records, 22 studies (3483 patients; 2706 RVOT, 777 LVOT) evaluating 21 ECG algorithms were included. The ‘Weighted hybrid score’ algorithm showed the highest diagnostic accuracy (S=21.2 (0.3, 39.0); DOR=275.8 (7.1, 1642.5)), with pooled sensitivity of 0.83 (0.53, 0.98) and specificity of 0.92 (0.68, 0.99). Conversely, the ‘Earliest onset or peak/nadir in lead V2’ algorithm had the lowest accuracy.

**Conclusions:**

Among existing ECG algorithms, the ‘Weighted hybrid score’ demonstrates superior diagnostic performance for differentiating LVOT from RVOT arrhythmias and is recommended for clinical application.

**PROSPERO registration number:**

CRD42024567531.

**WHAT IS ALREADY KNOWN ON THIS TOPIC**

- Several ECG algorithms are used to localise outflow tract ventricular arrhythmias (OTVAs), but their comparative accuracy is uncertain.

**WHAT THIS STUDY ADDS**

- In this network meta-analysis, we identified and compared 21 ECG algorithms; the ‘Weighted hybrid score’ had the highest diagnostic accuracy.
- The algorithm integrates ECG features with basic clinical parameters, making it practical and robust across various patient populations.

**HOW THIS STUDY MIGHT AFFECT RESEARCH, PRACTICE OR POLICY**

- The ‘Weighted hybrid score’ is clinically practical and may improve pre-ablation planning.
- These findings also lay the foundation for developing artificial intelligence-assisted ECG interpretation tools and further validation studies.

## Introduction

Ventricular arrhythmia (VA) is a common arrhythmia in clinical practice. Outflow tract ventricular arrhythmia (OTVA) is the most frequent type of idiopathic ventricular arrhythmia. Radiofrequency catheter ablation (RFCA) is a curative treatment for OTVA, which can be classified as either left ventricular outflow tract ventricular arrhythmia (LVOT VA) and right ventricular outflow tract ventricular arrhythmia (RVOT VA).^1^ The ECG-guided localisation of OTVA fulfils a crucial role in optimising procedural aspects, including vascular access, catheter selection, anticoagulation management and procedure duration, as well as for helping to minimise complication risks.^2 3^ However, the ECG morphologies of LVOT VA and RVOT VA are often similar, indicative of their close anatomical relationship. QRS morphology is characterised by left bundle branch block (R/S-wave amplitude ≤1 in lead V1) and inferior axis (positive polarity in all inferior leads).^4 5^ A series of ECG algorithms, therefore, have been developed to distinguish LVOT VA from RVOT VA.^1 6^

Although numerous ECG algorithms are available for differentiating LVOT VA from RVOT VA, there remains little consensus on which algorithm is optimal. The gold standard for this differentiation is the outcome of RFCA.^7 8^ Comparing the validity of individual ECG algorithms can help identify the relative diagnostic accuracy of each algorithm and guide clinicians in selecting the most appropriate algorithm. To our knowledge, no previous systematic review has assessed the diagnostic test accuracy of ECG algorithms for differentiating OTVA origins, and the most accurate ECG algorithm remains unknown. Diagnostic test accuracy network meta-analysis (DTA-NMA) offers several advantages: (1) it enables comparisons of diagnostic accuracy between tests that have not been directly compared; (2) it ranks all available tests based on their diagnostic performance by synthesising evidence from a network of studies; and (3) it enhances precision by incorporating all available data into a single model.^9 10^ Therefore, the aim of this systematic review and DTA-NMA was to identify the ECG algorithm with the highest diagnostic accuracy for differentiation between LVOT VA and RVOT VA.

## Methods

We conducted a systematic review and NMA of DTA studies to synthesise varied sources of evidence from a network of studies comparing differing ECG algorithms for differentiating LVOT VA from RVOT VA. This review followed the Preferred Reporting Items for Systematic Reviews and Meta-Analysis of Diagnostic Test Accuracy Studies (PRISMA-DTA)^11^ (onlinesupplemental additional files 1  2) and the PRISMA extension statement for NMA (PRISMA-NMA)^12^ (online supplemental additional file 3).

### Protocol

The review protocol was developed in accordance with the Cochrane Handbook for Systematic Reviews of Diagnostic Test Accuracy and PRISMA 2020 statement.^13^ It was registered in PROSPERO (CRD42024567531).

### Search strategy

We searched MEDLINE, EMBASE and the Cochrane Central Register of Controlled Trials for observational studies (both retrospective and prospective) published between 1 January 1990 and 7 May 2025, which evaluated ECG algorithms for distinguishing LVOT VA from RVOT VA. The search strategy was developed and adapted for each database (online supplemental appendix 1).

### Study selection

Inclusion criteria were as follows: (1) adult patients with idiopathic OTVA who underwent successful RFCA, defined as the absence of spontaneous or inducible clinical OTVA with or without isoproterenol infusion at the end of the ablation procedure; (2) full-text observational studies published in English, comparing two or more ECG algorithms for differentiating LVOT VA from RVOT VA; (3) studies with available 2×2 data (ie, true positives (TP), true negatives (TN), false negatives (FN), false positives (FP)) for analysis. If tabulated data were not directly reported, we back-calculated TP, TN, FN and FP values from the reported estimates of sensitivity, specificity, positive predictive value, negative predictive value and total number of patients. Exclusion criteria were as follows: (1) studies that did not provide sufficient information to extract or calculate TP, FP, TN and FN values; (2) protocols, review articles, conference abstracts, guidelines, consensus, comments, letters and brief reports; (3) duplicate publications; (4) studies evaluating ECG algorithms not based on the standard 12-lead ECG; (5) studies focused solely on the localisation of subsites within the RVOT or LVOT. Two review authors (ZH and ML) independently, and in duplicate, screened article titles, abstracts and full texts to identify eligible studies.

### Data collection, data extraction, risk of bias and applicability

One review author (ZH) extracted data using a predefined form, and another reviewer (ML) independently verified the extraction. The form was pilot tested with five full-text articles before data abstraction. We extracted 2×2 classification tables and data on: participant, ECG algorithm and study characteristics, as detailed in table 1. Two review authors (ZH and ML) independently assessed methodological quality using the Quality Assessment of Diagnostic Accuracy Studies (QUADAS-2) for patient selection, ECG algorithm, reference standard, flow and timing.^14^ A study was deemed to have a high overall risk of bias if any domain was rated as high risk. Applicability concerns for patient selection, ECG algorithm and reference standard were also evaluated.^14^ Disagreements in study selection, data extraction or methodological quality assessment were settled through discussion, or by consulting a third review author as an arbitrator.

**Table 1:** Characteristics for data abstraction

Group	Data abstracted
Participant characteristics	Age; proportion of females
ECG algorithms characteristics	Formular; reference standard; TP, TN, FP, FN; sensitivity, specificity, positive predicted value, negative predictive value with corresponding uncertainty measures (eg, 95% CI)
Study characteristics	Year of publication; journal name; country where study completed; study design; setting of participant recruitment; study duration; study setting; sample size

FN, false negatives; FP, false positives; TN, true negatives; TP, true positives.

### Data analysis methods

To assess the diagnostic accuracy of each ECG algorithm individually, we used the 2×2 table of the individual studies as defined by the results of the ECG algorithm against the reference standard. When the eligible studies formed a network of ECG algorithms, a DTA-NMA was conducted to incorporate all available evidence in a single model. A reference standard defines LVOT origin or RVOT origin, and a network comprises ECG algorithms only.^15^ We performed a Bayesian DTA-NMA model comparing the 2×2 table of the results of each ECG algorithm against the reference standard, accounting for correlation between algorithms from the same study (online supplemental appendix 2).^16^ Superiority index (S)^16 17^ was calculated within the DTA-NMA to rank diagnostic performances of ECG algorithms, whereby the higher the S, the better the ECG algorithm.

We assumed the transitivity assumption for the NMA would be satisfied, as the included studies would be similar in patient population, cut-off value definitions, whereby the conduct of ECG algorithms and reference tests were performed at the same time point on the diagnostic test pathway.^18^ We estimated sensitivity and specificity for each ECG algorithm along with their 95% CIs and between-test and between-study heterogeneity.^10^ Heterogeneity among independent studies was evaluated using the *I*² statistic. More specifically, *I*² values <50% suggested low heterogeneity. In contrast, *I*² values ≥50% indicated moderate and ≥75% considerable heterogeneity, warranting sensitivity analysis.^19 20^ A consistency test was not conducted (direct evidence is expected to be equivalent to indirect evidence) in this DTA-NMA as the specified approaches used in the NMA of interventions could not be applied in this study.^18 21^

We performed the DTA-NMA using the *rstan* package in R V.4.3.1^10 22^ and code provided by Nyaga *et al*.^16^ These authors developed an analysis of variance (ANOVA) model to perform Bayesian network meta-analysis of diagnostic test accuracy, where the core concept compares the accuracy of different diagnostic tests by calculating the relative ratios of diagnostic performance measures. The model relies on a two-way ANOVA structure with two independent binomial distributions to describe TP and TN for patients and healthy subjects respectively, while accounting for the correlation between sensitivity and specificity.^16^

## Results

### Literature search and study characteristics

**Figure 1:**
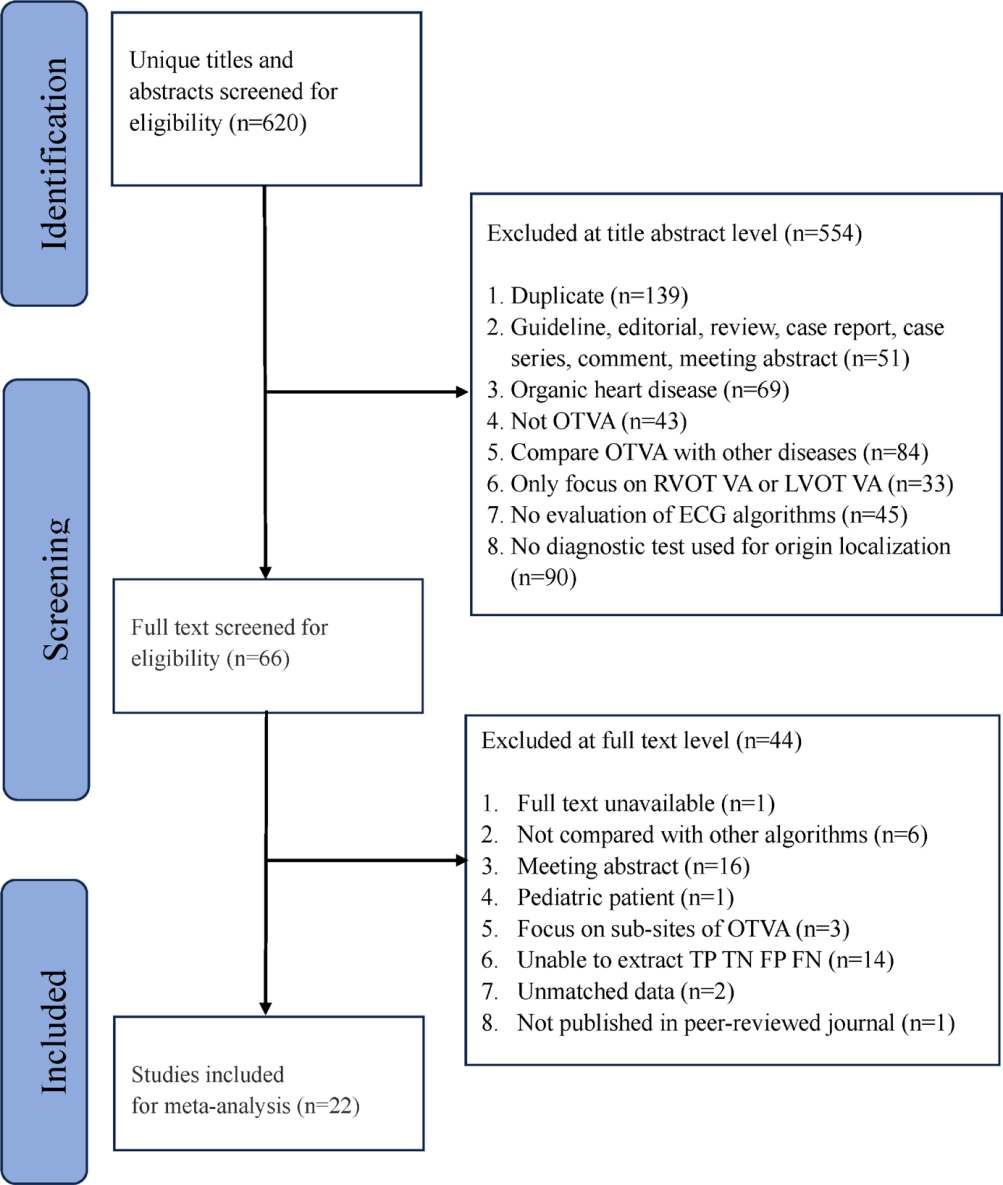
PRISMA (Preferred Reporting Items for Systematic Reviews and Meta-Analyses) flow diagram of the studies selection process. FN, false negatives; FP, false positives; LVOT VA, left ventricular outflow tract ventricular arrhythmia; OTVA, outflow tract ventricular arrhythmia; RVOT VA, right ventricular outflow tract ventricular arrhythmia; TN, true negatives; TP, true positives.

**Figure 2:**
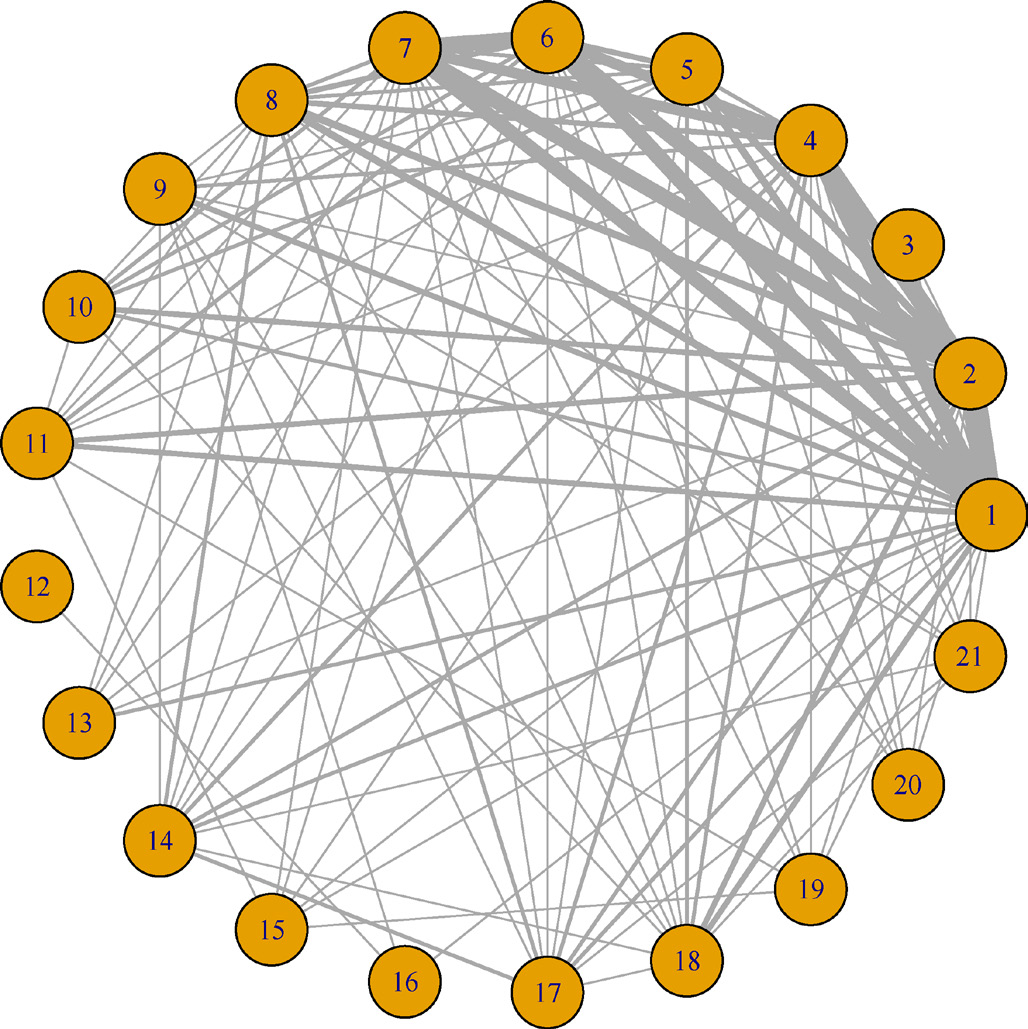
Network plot of primary studies assessing the diagnostic accuracy of two or more ECG algorithms. Nodes represent the ECG algorithms being compared, and lines represent the available direct comparisons (comparisons evaluated in at least one study) between pairs of ECG algorithms. Lines are weighted according to the numbers involved in direct comparison. 1, TZI; 2, V2S/V3R; 3, TZI+V2 S/V3R; 4, V2 transition ratio; 5, S-R difference in V1 and V2; 6, R/S amplitude index; 7, R-wave duration index; 8, Combined index; 9, V2 R-wave duration index+R/S amplitude index; 10, ISA; 11, R amplitude in lead I; 12, Clinical score; 13, Corrected TZI; 14, Earliest onset or peak/nadir in lead V2; 15, Hybrid algorithm; 16, R/S transition; 17, RWDI; 18, V2QRS_i40_; 19, Weighted hybrid score; 20, RV1+RV3 index; 21, Multistep algorithm. ISA, initial r wave surface area; RWDI, R-wave deflection interval; TZI, transitional zone index.

Of the total 620 records screened, 22 studies^36 23^,^42^ (3483 participants; 5 prospective studies) were included in the analysis (figure 1; online supplemental appendix 3). These studies evaluated 21 different ECG algorithms in total (online supplemental appendix4), with each individual study assessing between 2 and 10 algorithms. The most frequently assessed ECG algorithms in the included studies were the ‘transitional zone index (TZI)’ and ‘V2S/V3R index’, investigated in 18 (82%) and 17 (77%) studies, respectively. The least frequently assessed algorithms were: ‘TZI+V2 S/V3R’, ‘Clinical score’, ‘Hybrid algorithm’, ‘Weighted hybrid score’, ‘RV1+RV3 index’ and ‘Multistep algorithm’; each of which appeared in only one study. All studies included in this analysis reported acute procedural success. In addition, 17 studies further evaluated long-term freedom from recurrence, with 12 employing a minimum follow-up of 3 months and 5 applying a follow-up of at least 6 months. Although not all studies reported long-term follow-up, those that did assessed recurrence based on the reappearance of OTVA during follow-up, typically through Holter monitoring or clinical evaluation. Among the 3483 participants, 2706 underwent successful ablation at RVOT and 777 at LVOT. Study sample sizes ranged from 29^38^ to 695^39^ participants. The mean age of participants ranged from 41^31^ to 52 years.^3 24^ The proportion of female participants ranged from 45%^38^ to 76%.^42^ The studies were conducted across 10 countries, with the majority originating from China (10 studies, 45%) and 3 (14%) studies from the USA. The NMA comprised 21 nodes and 107 edges, visually represented in figure 2.

### Methodological quality of included studies

**Figure 3:**
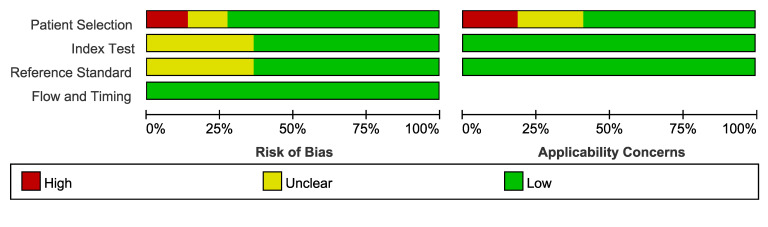
Assessment of the risk of bias (left) and applicability concerns (right) for the included test accuracy studies using the QUADAS-2 (Quality Assessment of Diagnostic Accuracy Studies) tool.

Overall, the risk of bias varied across studies, with 12 studies^36 28 31 35^,^42^ at low risk of bias, 7 studies at moderate risk and the remaining 3 studies^26 29 34^ at high risk. Figure 3 summarised the methodological quality of the 22 included studies, with the main source of bias from two aspects: (1) the lack of blinding between ECG algorithms and reference standard; and (2) the inclusion of patients with a right bundle branch block (RBBB) pattern in OTVA. There were no concerns regarding the risk of bias related to the flow and timing of tests, as well as applicability concerns for index test or reference standard (figure 3).

### Network meta-analysis

**Figure 4:**
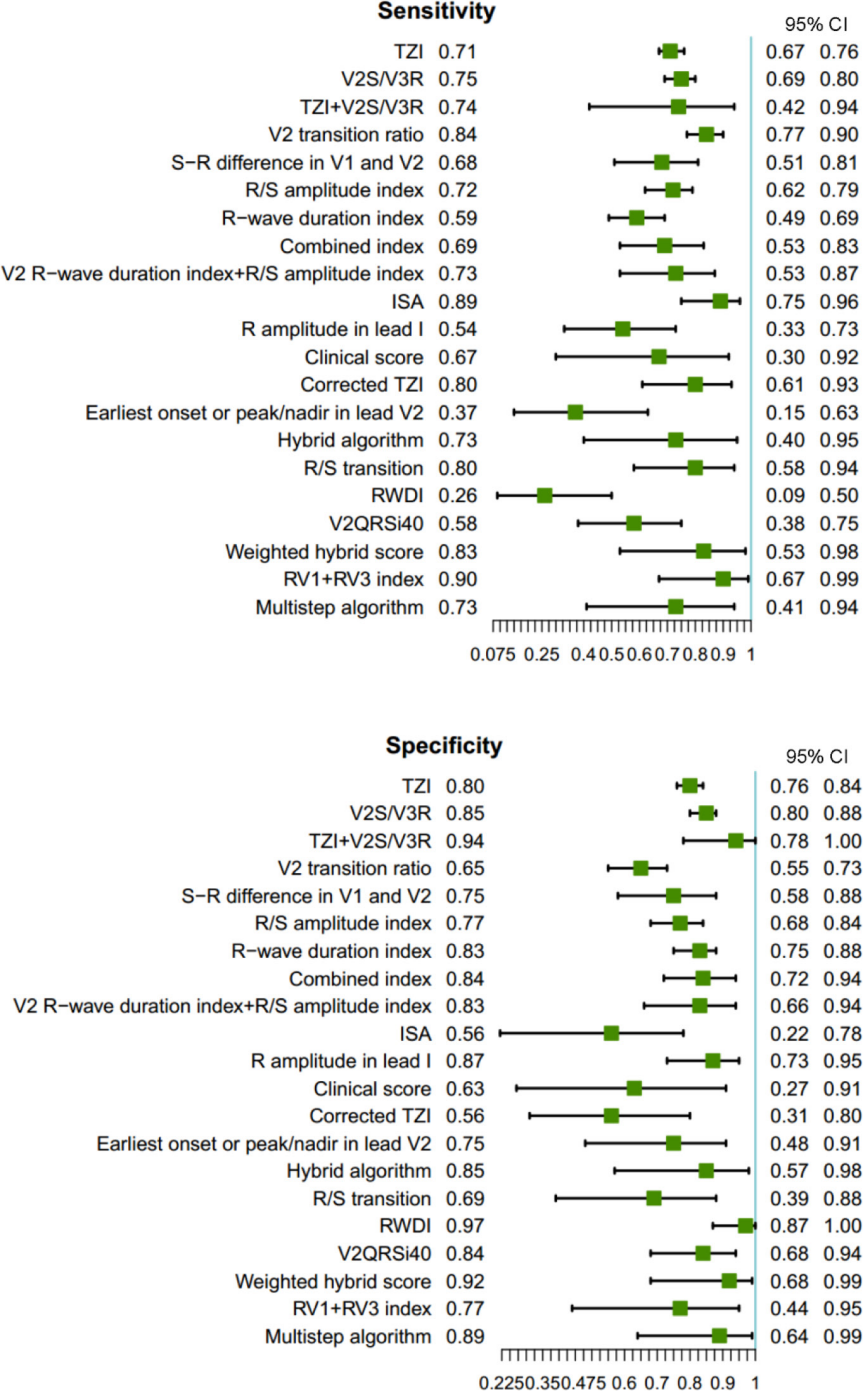
Network meta-analysis forest plots for (above) sensitivity and (below) specificity of ECG algorithms. ISA, initial r wave surface area; RWDI, R-wave deflection interval; TZI, Transitional Zone Index.

We conducted a DTA-NMA including all eligible ECG algorithms. Our NMA suggested that the ‘Weighted hybrid score’ algorithm was associated with highest S (21.2 (0.3, 39.0) and DOR (275.8 (7.1, 1642.5)), with sensitivity (0.83 (0.53, 0.98)) and specificity (0.92 (0.68, 0.99)). According to the S ranking, this algorithm was followed by the ‘TZI+V2 S/V3R’ algorithm (sensitivity: 0.74 (0.42, 0.94); specificity 0.94 (0.78, 1.00); S: 16.8 (0.3, 39.0); DOR: 235.9 (6.6, 1367.6)). The ‘Earliest onset or peak/nadir in lead V2’ algorithm (sensitivity: 0.37 (0.15, 0.63); specificity 0.75 (0.48, 0.91); S: 0.1 (0.0, 0.6); DOR: 2.5 (0.3, 9.3)) ranked lowest (online supplemental appendix 5). Overall, six ECG algorithms (V2 transition ratio, initial r wave surface area (ISA), Corrected TZI, R/S transition, Weighted hybrid score, RV1+RV3 index) were associated with a sensitivity of ≥0.80, and three (TZI+V2 S/V3R, R-wave deflection interval (RWDI), Weighted hybrid score) with a specificity of ≥0.90 (figure 4). Relative accuracy of all ECG algorithms compared with the ‘Weighted hybrid score’ is shown in online supplemental appendix 6. Notably, the ‘V2S/V3R index, Corrected TZI and R/S transition’ exhibited similar sensitivity to the ‘Weighted hybrid score’. The ‘TZI+V2 S/V3R, Combined index, R amplitude in lead I, Hybrid algorithm and Multistep algorithm’ had comparable specificity.

The global *I*² values for sensitivity and specificity were 1.67% and 56.58%, respectively, indicating low heterogeneity for sensitivity and moderate heterogeneity for specificity. The low *I*² value for sensitivity suggests high consistency across the included studies in identifying true positive cases, that is, LVOT origin, with variability more likely due to random error rather than substantive differences. For specificity, which reflects the ability to correctly identify true negative cases (ie, RVOT origin), the *I*² value exceeds the 50% threshold, suggesting that variability in diagnosing RVOT origin was more pronounced. The higher *I*² for specificity suggests potential influence from methodological, technical or population-related differences across studies. Nevertheless, this level of heterogeneity is generally considered acceptable, and the pooled estimates for both sensitivity and specificity can still be interpreted with reasonable confidence.^19 20^ To explore the borderline heterogeneity in specificity further, we conducted subgroup analyses to assess the robustness of our findings.

### Sensitivity analysis

Sensitivity analyses, such as a leave-one-out analysis, could further assess robustness; however, the limited number of studies for several algorithm pairs and the structure of the DTA-NMA restricted its feasibility. Attempting such analyses could have disrupted the closed-loop structure required for valid network meta-analysis, potentially undermining the integrity of the evidence synthesis.^43^ As an alternative, we conducted subgroup analyses excluding the study that explicitly involved RBBB cases and also performed a subgroup analysis restricted to studies that evaluated long-term RFCA success.

First, we excluded studies that included patients with OTVA exhibiting RBBB morphology. The ‘Weighted hybrid score’ continued to yield the highest S (21.7 (1.0, 35.0)), reinforcing its superior overall diagnostic performance even when RBBB cases were removed (online supplemental appendix 7). Notably, the heterogeneity in specificity did not decrease following this exclusion, suggesting that the inclusion of RBBB patients is unlikely to be a major source of inconsistency across studies. This supports the generalisability of the ‘Weighted hybrid score’ to OTVA cases irrespective of their ECG morphology, including those with RBBB patterns.

Additionally, we performed a subgroup analysis restricted to studies that defined RFCA success based not only on acute suppression of OTVA but also on long-term freedom from recurrence. In this subgroup, the ‘Weighted hybrid score’ again demonstrated the highest S (22.0 (0.6, 39.0)), reinforcing its reliability under more stringent clinical criteria. Interestingly, the S for the ‘ISA’ algorithm improved substantially (S=18.0 (0.2, 37.0)), suggesting that its diagnostic value may be more evident in studies with longer-term outcome assessments. The ‘R-wave duration index’ and the ‘Earliest onset or peak/nadir in lead V2’ demonstrated similarly low diagnostic accuracy, as reflected by identical S (S=0.2) (online supplemental appendix 7). These findings suggested that both these two algorithms performed comparably and were among the least effective within this subgroup.

## Discussion

Our systematic review and DTA-NMA of 21 ECG algorithms for differentiating LVOT VA from RVOT VA, based on 22 studies, involving 3483 patients with OTVA who underwent RFCA, identified the ‘Weighted hybrid score’ algorithm as having the highest diagnostic accuracy, while the ‘Earliest onset or peak/nadir in lead V2’ algorithm ranked the lowest. Subgroup analyses further confirmed the consistent and robust performance of the ‘Weighted hybrid score’ across various patient populations and clinical definitions of ablation success. The ‘Weighted hybrid score’ incorporates five clinically accessible variables (R/S transition in OTVA, V3 R-wave amplitude, age, gender and hypertension history^36^), making it practical for routine clinical use. In comparison, the ‘Earliest onset or peak/nadir in lead V2’, previously validated with poor sensitivity and moderate specificity,^42^ yielded similarly limited diagnostic value in this DTA-NMA. Our analysis showed that the ‘RV1+RV3 index’ demonstrated the highest sensitivity, while the ‘RWDI’ had the lowest. Additionally, the ‘RWDI’ demonstrated the highest specificity, while the ‘ISA’ and ‘Corrected TZI’ had the lowest (figure 4).

The prevalence of LVOT (10–47%) in our study aligns with previously reported ranges (12–45%).^8 44 45^ This analysis showed that several algorithms met the criteria for being ‘useful’ (sensitivity+specificity being ≥1.5), including ‘TZI, V2S/V3R, TZI+V2 S/V3R, Combined index, V2 R-wave duration index+R/S amplitude index, Hybrid algorithm, Weighted hybrid score, RV1+RV3 index, and Multistep algorithm’.^46^ Notably, the ‘Weighted hybrid score’ was the only algorithm to meet the criteria for ‘good validity’ (sensitivity and specificity ≥0.8).^47 48^ To situate our findings into the clinical context, ‘Weighted hybrid score’ would identify 297 positive cases in a population of 1000 patients with OTVA and an LVOT VA prevalence of 29%, whereby 240 cases would be correctly identified, 57 would be falsely identified and 50 would be missed.

Although moderate heterogeneity was observed for specificity, heterogeneity for sensitivity (true positive rate) remained low. This distinction is critical, as the primary aim of this DTA-NMA was to evaluate the diagnostic accuracy of ECG algorithms in identifying LVOT origin, thereby minimising FN. The consistent sensitivity reinforces the robustness of the findings in detecting true LVOT cases. While variability in specificity reflects some inconsistency in distinguishing RVOT origins, this is acceptable given the primary clinical objective of accurately identifying LVOT cases. Therefore, the moderate heterogeneity in specificity does not substantially compromise the reliability or clinical utility of the NMA conclusions.

The accuracy and practicality of existing ECG algorithms have notable limitations. First, several ECG algorithms, especially the two most frequently compared, ‘TZI’ and ‘V2S/V3R’, have demonstrated inconsistent discriminative performance in subsequent studies compared with their original publications.^628 31^,^33 37 38 42^ Second, some ECG algorithms, such as the ‘S-R difference in V1 and V2’, ‘ISA’, ‘R/S amplitude index’ and ‘R-duration index’, were derived from limited ECG patterns, often relying solely on leads V1 and V2.^33 49 50^ Additionally, the practical application of certain morphological algorithms is challenging. For example, the ‘V2QRS_i40_’ requires calculating the amplitude of the QRS complex within the initial 40 ms in lead V2, which may vary substantially and reduce its clinical applicability.^6^ Moreover, most algorithms were developed using small sample sizes and have not been validated in ethnically diverse populations.^2 28 33 50^ Other factors, such as cardiac rotation, lead position, ventricular hypertrophy, chest wall thickness (eg, obesity, chest wall deformity) and preferential conduction, may further compromise diagnostic accuracy.^1 27^ In contrast, the ‘Weighted hybrid score’ integrates clinical and ECG parameters, while accounting for the effects of cardiac rotation.

From a clinical perspective, accurate identification of OTVA origin is critical. On the one hand, selecting the appropriate vascular access before RFCA minimises procedural time and complications. On the other hand, according to the 2022 ESC guideline and the 2019 HRS/EHRA/APHRS/LAHRS consensus,^7 8^ catheter ablation is recommended as a first-line treatment in symptomatic patients with frequent RVOT VA, given its high success rate (80–95%) and low complication rate. Conversely, for patients with symptomatic idiopathic LVOT VA, antiarrhythmic medications (eg, beta-blockers or non-dihydropyridine calcium channel blockers) are generally preferred over catheter ablation. Therefore, accurately distinguishing the LVOT from RVOT origin is crucial for guiding appropriate treatment decisions, particularly regarding ablation indications. In addition, ECG can be chosen to develop a diagnostic model due to its non-invasive, widely used and cost-effective nature.^51^ To ensure ECG algorithms are valid, studies focused on the quality of design and accuracy of diagnostic tests should be improved, ensuring that the results of ECG algorithms are blind to clinicians conducting the RFCA reference standard, enrolling patients only with left bundle branch block pattern in OTVA, and confirming RFCA success through follow-up assessments. Future studies should focus on integrating the strengths of various algorithms, leveraging artificial intelligence and machine learning technologies to develop more accurate and automated diagnostic tools. Large-scale, multicentre validation across diverse populations is also essential to improve generalisability and robustness. Additionally, linking electrophysiological findings with anatomical characteristics features as a way to enhance localisation accuracy and guide ablation strategies. Finally, developing dynamic diagnostic tools that are capable of capturing the temporal variability of ectopic activity in the outflow tract may also advance this field.

Previous systematic reviews have summarised ECG algorithms for differentiation between LVOT VA and RVOT VA.^1 52^ However, this DTA-NMA is the first to integrate all available evidence into a single comparative model. Because no single study can capture the full performance spectrum of all tests, our comprehensive approach offers a more reliable basis for clinical practice and policy decision-making. By evaluating multiple ECG algorithms across diverse settings, including both high-resource and low-resource environments, our findings offer enhanced generalisability and relevance.

### Limitations

This DTA-NMA has some limitations. First, the inclusion of both prospective and retrospective studies introduces potential methodological heterogeneity. Of the 22 included studies, 17 were retrospective, and much of the evidence was derived from retrospective observational studies, which are inherently subject to biases such as incomplete data, lack of blinding and inconsistent outcome definitions.^53^ Second, our study focused solely on ECG algorithms derived from standard 12-lead ECG, excluding those based on 18-lead ECG. However, since the 12-lead ECG is the most commonly used and cost-effective non-invasive ECG in clinical practice, our findings remain applicable across various healthcare settings.

Another important limitation lies in the use of the successful ablation site as the reference standard for determining the origin of OTVA. Although this definition is clinically practical, it may not always reflect the true site of origin. Some arrhythmia foci may be located in the contiguous epicardium or intramural segments between the RVOT and LVOT, where ablation at an anatomically adjacent site may be sufficient to eliminate the arrhythmia.^6 37 42^ This spatial proximity may lead to misclassification of the true origin, introducing a degree of uncertainty in evaluating the diagnostic accuracy of ECG algorithm. This potential discrepancy may also help explain a few cases where the ECG algorithm results did not align with the final localisation.^37^ Additionally, some studies have made direct comparisons between different ECG algorithms within the same populations in which a novel algorithm was originally developed, without independent external validation. This may lead to overestimation of diagnostic performance, and the reproducibility and clinical applicability of these algorithms remain uncertain.^54 55^

Lastly, there was substantial variation in RVOT and LVOT sample sizes across studies, reflecting their differing clinical prevalence. LVOT cases ranged from 8 to 100, while RVOT cases ranged from 21 to 625, with greater variability in the RVOT group. Since specificity reflects the ability to correctly identify true negative cases (ie, RVOT origins), this imbalance may have contributed to the observed heterogeneity in specificity estimates. Moreover, variability in study quality, population characteristics and algorithm implementation likely further contributes to between-study heterogeneity. Therefore, while this DTA-NMA offers valuable comparative insights, the findings should be interpreted with caution, particularly when applied across diverse clinical contexts.

## Conclusions

This systematic review and DTA-NMA demonstrated that the ‘Weighted hybrid score’ has the highest diagnostic accuracy among 21 ECG algorithms for differentiation between LVOT VA and ROVT VA. Given its simplicity and clinical utility, the implementation of the ‘Weighted hybrid score’ is recommended to assist in localising OTVA prior to RFCA, thereby informing both the indication for ablation and the choice of vascular access.

## Supplementary material

10.1136/heartjnl-2025-325916 online supplemental file 1

10.1136/heartjnl-2025-325916 online supplemental file 2

10.1136/heartjnl-2025-325916 online supplemental file 3

10.1136/heartjnl-2025-325916 online supplemental file 4

## Data Availability

Data are available upon reasonable request.
