# Supplementary material for: Diagnostic accuracy of electrocardiogram algorithms for differentiating left from right outflow tract ventricular arrhythmia: a systematic review and network meta-analysis
Source: Heart. 2025 Jul 3;112(3):e325916. doi: 10.1136/heartjnl-2025-325916 (PMC12911587; doi:10.1136/heartjnl-2025-325916)
Supplement: online supplemental file 4 [file heartjnl-112-3-s004.docx]

**Appendix 1. Search strategies in different databases**

Medline search strategy: 227 studies found from January 1990 to May 7, 2025

(("receiver operating characteristic"[Text Word] OR "roc curve"[MeSH Terms] OR ("sensitivity"[Text Word] OR "specificity"[Text Word] OR "predictive value"[Text Word] OR "sensitivity and specificity"[MeSH Terms])) AND ((("arrhythmias, cardiac"[MeSH Terms] OR "tachycardia"[MeSH Terms] OR "tachycardia, ventricular"[MeSH Terms] OR (("heart ventricles"[MeSH Terms] OR ("heart"[All Fields] AND "ventricles"[All Fields]) OR "heart ventricles"[All Fields] OR "ventricular"[All Fields] OR "ventricularization"[All Fields] OR "ventricularized"[All Fields]) AND "arrhythmias, cardiac"[MeSH Terms]) OR "ventricular premature complexes"[MeSH Terms] OR "ventricular premature complexes"[MeSH Terms] OR "ventricular premature complexes"[MeSH Terms]) AND ("aortic sinus cusp"[Text Word] OR "outflow tract"[Text Word])) OR ((("outflow"[All Fields] OR "outflowing"[All Fields] OR "outflows"[All Fields]) AND ("tract"[All Fields] OR "tract s"[All Fields] OR "tracts"[All Fields]) AND ("heart ventricles"[MeSH Terms] OR ("heart"[All Fields] AND "ventricles"[All Fields]) OR "heart ventricles"[All Fields] OR "ventricular"[All Fields] OR "ventricularization"[All Fields] OR "ventricularized"[All Fields])) AND "arrhythmias, cardiac"[MeSH Terms])) AND "electrocardiography"[MeSH Terms]) AND ((english[Filter]) AND (1990:2025[pdat]))

EMBASE search strategy: 376 studies found from January 1990 to May 7, 2025

#1 'outflow tract'/exp OR 'outflow tract'

#2 'outflow tract ventricular tachycardia'/exp OR 'outflow tract ventricular tachycardia'

#3 'outflow tract ventricular arrhythmia'/exp OR 'outflow tract ventricular arrhythmia'

#4 'heart ventricle arrhythmia'/exp OR 'heart ventricle arrhythmia'

#5 'heart ventricle tachycardia'/exp OR 'heart ventricle tachycardia'

#6 'heart ventricle extrasystole'/exp OR 'heart ventricle extrasystole'

#7 'premature ventricular complex'/exp OR 'premature ventricular complex'

#8 #4 OR #5 OR #6 OR #7

#9 #1 AND #8

#10 #2 OR #3 OR #9

#11 'premature ventricular complex':ti,ab,kw OR 'premature ventricular contraction':ti,ab,kw OR 'premature ventricular beat':ti,ab,kw OR 'ventricular tachycardia':ti,ab,kw OR 'ventricular arrhythmia':ti,ab,kw

#12 'outflow tract':ti,ab,kw OR 'aortic sinus cusp':ti,ab,kw

#13 #11 AND #12

#14 #10 OR #13

#15 'sensitivity and specificity'/exp OR 'sensitivity and specificity'

#16 'predictive value'/exp OR 'predictive value'

#17 'receiver operating characteristic'/exp OR 'receiver operating characteristic'

#18 sensitivity:ti,ab,kw OR specificity:ti,ab,kw OR 'predictive value':ti,ab,kw OR 'receiver operating characteristic':ti,ab,kw

#19 #15 OR #16 OR #17

#20 #18 OR #19

#21 #14 AND #20

#22 'electrocardiogram'/exp OR 'electrocardiogram'

#23 'electrocardiography'/exp OR 'electrocardiography'

#24 electrocardiogram:ti,ab,kw OR electrocardiography:ti,ab,kw

#25 #22 OR #23 OR #24

#26 #21 AND #25

#27 #21 AND #25 AND [english]/lim

#28 #21 AND #25 AND [english]/lim AND [humans]/lim

#29 #21 AND #25 AND [english]/lim AND [humans]/lim AND [1990-2025]/py

Cochrane library search strategy: 15 studies found from January 1990 to May 7, 2025

#1 MeSH descriptor: [Ventricular Premature Complexes] explode all trees

#2 MeSH descriptor: [Tachycardia, Ventricular] explode all trees

#3 MeSH descriptor: [Arrhythmias, Cardiac] explode all trees

#4 #1 OR #2 OR #3

#5 (outflow tract):ti,ab,kw (Word variations have been searched)

#6 (aortic sinus cusp):ti,ab,kw (Word variations have been searched)

#7 (outflow tract ventricular arrhythmia):ti,ab,kw (Word variations have been searched)

#8 ("ventricular outflow tract"):ti,ab,kw (Word variations have been searched)

#9 #5 OR #6 OR #8

#10 #4 AND #9

#11 #7 OR #10

#12 MeSH descriptor: [Sensitivity and Specificity] explode all trees

#13 (Sensitivity):ti,ab,kw OR ("specificity"):ti,ab,kw OR (predictice value):ti,ab,kw OR ("receiver operating characteristic curve"):ti,ab,kw (Word variations have been searched)

#14 MeSH descriptor: [ROC Curve] explode all trees

#15 MeSH descriptor: [Predictive Value of Tests] explode all trees

#16 #12 OR #14 OR #15

#17 #16 OR #13

#18 #17 AND #11

Records identified from citation search: 2 studies

1. Betensky BP, Park RE, Marchlinski FE, et al. The V(2) transition ratio: a new electrocardiographic criterion for distinguishing left from right ventricular outflow tract tachycardia origin. J Am Coll Cardiol 2011;57:2255-62.
2. Ouyang F, Fotuhi P, Ho SY, et al. Repetitive monomorphic ventricular tachycardia originating from the aortic sinus cusp: electrocardiographic characterization for guiding catheter ablation. J Am Coll Cardiol 2002;39:500-8.

**Appendix 2. List of included studies**

1 Zhang F, Hamon D, Fang Z, et al. Value of a Posterior Electrocardiographic Lead for Localization of Ventricular Outflow Tract Arrhythmias: The V4/V8 Ratio. JACC: Clinical Electrophysiology 2017;3:678-86.

2 Bourquin L, Küffer T, Asatryan B, et al. Validation of a clinical model for predicting left versus right ventricular outflow tract origin of idiopathic ventricular arrhythmias. PACE - Pacing and Clinical Electrophysiology 2023;46:1186-96.

3 Cheng D, Ju W, Zhu L, et al. V3R/V7 index a novel electrocardiographic criterion for differentiating left from right ventricular outflow tract arrhythmias origins. Circulation: Arrhythmia and Electrophysiology 2018;11.

4 Gabriels JK, Abdelrahman M, Nambiar L, et al. Reappraisal of electrocardiographic criteria for localization of idiopathic outflow region ventricular arrhythmias. Heart Rhythm 2021;18:1959-65.

5 Celikyurt U, Agir A, Karauzum I, et al. Predicting value of coupling interval variability in determining the origin of ventricular premature contractions with V3 transition. Journal of Interventional Cardiac Electrophysiology 2018;53:169-74.

6 Lin C, Zheng C, Zhou DP, et al. Origins location of the outflow tract ventricular arrhythmias exhibiting qrS pattern or QS pattern with a notch on the descending limb in lead V1. BMC Cardiovascular Disorders 2017;17.

7 Yoshida N, Inden Y, Uchikawa T, et al. Novel transitional zone index allows more accurate differentiation between idiopathic right ventricular outflow tract and aortic sinus cusp ventricular arrhythmias. Heart Rhythm 2011;8:349-56.

8 Anderson RD, Kumar S, Binny S, et al. Modified Precordial Lead R-Wave Deflection Interval Predicts Left- and Right-Sided Idiopathic Outflow Tract Ventricular Arrhythmias. JACC Clin Electrophysiol 2020;6:1405-19.

9 Xie S, Kubala M, Liang JJ, et al. Lead I R-wave amplitude to differentiate idiopathic ventricular arrhythmias with left bundle branch block right inferior axis originating from the left versus right ventricular outflow tract. J Cardiovasc Electrophysiol 2018;29:1515-22.

10 Waight MC, Li AC, Leung LW, et al. Hourly variability in outflow tract ectopy as a predictor of its site of origin. J Cardiovasc Electrophysiol 2022;33:7-16.

11 Ludwik B, Deutsch K, Mazij M, et al. Electrocardiographic algorithms to guide the management strategy of idiopathic outflow tract ventricular arrhythmias. Pol Arch Intern Med 2017;127:749-57.

12 Jiao ZY, Li YB, Mao J, et al. Differentiating origins of outflow tract ventricular arrhythmias: A comparison of three different electrocardiographic algorithms. Brazilian Journal of Medical and Biological Research 2016;49.

13 He Z, Liu M, Yu M, et al. An electrocardiographic diagnostic model for differentiating left from right ventricular outflow tract tachycardia origin. J Cardiovasc Electrophysiol 2018;29:908-15.

14 Xia Y, Liu Z, Liu J, et al. Amplitude of QRS complex within initial 40 ms in V(2) (V(2)QRS(i40)): Novel electrocardiographic criterion for predicting accurate localization of outflow tract ventricular arrhythmia origin. Heart Rhythm 2020;17:2164-71.

15 Yoshida N, Yamada T, McElderry HT, et al. A novel electrocardiographic criterion for differentiating a left from right ventricular outflow tract tachycardia origin: the V2S/V3R index. J Cardiovasc Electrophysiol 2014;25:747-53.

16 Nikoo MH, Taheri S, Attar A. A novel ECG criterion to differentiate left from right ventricular outflow tract premature complex. Scandinavian Cardiovascular Journal 2020;54:139-45.

17 Zhang W, Huang K, Qu J, et al. A novel ECG algorithm to differentiate between ventricular arrhythmia from right versus left ventricular outflow tract. Journal of cardiovascular medicine (Hagerstown, Md) 2023;24:853-63.

18 Qiu S, Sun Z, Li X, et al. A novel and effective ECG method to differentiate right from left ventricular outflow tract arrhythmias: Angle-corrected V2S. Frontiers in Cardiovascular Medicine 2022;9.

19 Penela D, Falasconi G, Carreño JM, et al. A hybrid clinical and electrocardiographic score to predict the origin of outflow tract ventricular arrhythmias. Journal of Interventional Cardiac Electrophysiology 2023;66:1877-88.

20 Liu M, He Z, Ying P, et al. Differentiating Origins of Outflow Tract Ventricular Arrhythmias: The Correction of Transitional Zone Index Is Not Superior to the Original One. Cardiology 2024;149:137-46.

21 Chen N, Wang L, Jiao J, et al. RV1+RV3 Index to Differentiate Idiopathic Ventricular Arrhythmias Arising From Right Ventricular Outflow Tract and Aortic Sinus of Valsalva: A Multicenter Study. J Am Heart Assoc 2024;13:e033779.

22 Amadis MR, Lo LW, Salim S, et al. Multistep Algorithm to Predict RVOT PVC Site of Origin for Successful Ablation Using Available Criteria: A Two-Center Cross-Validation Study. Pacing Clin Electrophysiol 2025;48:128-36.

**Appendix 3. Characteristics of included studies**

| **Reference** | **ECG algorithm** | **Study**  **setting** | **Study duration** | **Mean age** | **Proportion of females** | **Sample size** | **LVOT** | **RVOT** | **TP** | **FN** | **TN** | **FP** |
| --- | --- | --- | --- | --- | --- | --- | --- | --- | --- | --- | --- | --- |
| **Prospective** | |  |  |  |  |  |  |  |  |  |  |  |
| Penela et al. 2023 [36] | Weighted hybrid score | Spain | Aug 2018 to Dec 2020 | 51.2±14.3 | 0.61 | 105 | 31 | 74 | 28 | 3 | 71 | 3 |
|  | Hybrid algorithm |  |  |  |  |  |  |  | 26 | 5 | 68 | 6 |
|  | V2S/V3R |  |  |  |  |  |  |  | 29 | 2 | 55 | 19 |
|  | TZI |  |  |  |  |  |  |  | 21 | 10 | 57 | 17 |
|  | V2 transition ratio |  |  |  |  |  |  |  | 27 | 4 | 60 | 14 |
|  | R-wave duration index |  |  |  |  |  |  |  | 17 | 14 | 70 | 4 |
|  | R/S amplitude index |  |  |  |  |  |  |  | 29 | 2 | 57 | 17 |
|  | R amplitude in lead I |  |  |  |  |  |  |  | 22 | 9 | 31 | 43 |
| Waight et al. 2021[38] | TZI | UK |  | 47.6±18.8 | 0.45 | 29 | 8 | 21 | 7 | 1 | 17 | 4 |
|  | V2S/V3R |  |  |  |  |  |  |  | 5 | 3 | 19 | 2 |
| Anderson et al. 2020 [42] | Combined index | Australia | May 2018 to March 2020 | 51.0±17.0 | 0.76 | 50 | 20 | 30 | 19 | 1 | 27 | 3 |
|  | V2S/V3R |  |  |  |  |  |  |  | 17 | 3 | 29 | 1 |
|  | R-wave duration index |  |  |  |  |  |  |  | 14 | 6 | 29 | 1 |
|  | R/S amplitude index |  |  |  |  |  |  |  | 16 | 4 | 24 | 6 |
|  | RWDI |  |  |  |  |  |  |  | 11 | 9 | 29 | 1 |
|  | V2 transition ratio |  |  |  |  |  |  |  | 17 | 3 | 22 | 8 |
|  | Earliest onset or peak/ nadir in lead V2 |  |  |  |  |  |  |  | 7 | 13 | 25 | 5 |
|  | TZI |  |  |  |  |  |  |  | 19 | 1 | 14 | 16 |
| Cheng et al. 2018[37] | TZI | China | Dec 2016 to Aug 2017 | 45.7±15.6 | 0.63 | 94 | 20 | 74 | 12 | 8 | 54 | 20 |
|  | V2 transition ratio |  |  |  |  |  |  |  | 17 | 3 | 49 | 25 |
|  | V2S/V3R |  |  |  |  |  |  |  | 17 | 3 | 61 | 13 |
| Zhang et al. 2017 [23] | V2 transition ratio | China | 2013-2015 | 44.0±16.0 | 0.65 | 40 | 12 | 28 | 8 | 4 | 19 | 9 |
|  | V2S/V3R |  |  |  |  |  |  |  | 4 | 8 | 20 | 8 |
| **Retrospective** | |  |  |  |  |  |  |  |  |  |  |  |
| Liu et al. 2024 [39] | TZI | China | Aug 2008 to Dec 2016 | 42.0±13.0 | 0.70 | 695 | 70 | 625 | 47 | 23 | 551 | 74 |
|  | Corrected TZI |  |  |  |  |  |  |  | 59 | 11 | 372 | 253 |
| Chen et al. 2024 [40] | R/S amplitude index | China | Mar 2018 to Dec 2021 | 45.3±16.4 | 0.60 | 150 | 40 | 110 | 27 | 13 | 107 | 3 |
|  | R-wave duration index |  |  |  |  |  |  |  | 37 | 3 | 40 | 70 |
|  | V2 transition ratio |  |  |  |  |  |  |  | 36 | 4 | 70 | 40 |
|  | TZI |  |  |  |  |  |  |  | 23 | 17 | 95 | 15 |
|  | V2S/V3R |  |  |  |  |  |  |  | 30 | 10 | 92 | 18 |
|  | S-R difference in V1-V2 |  |  |  |  |  |  |  | 32 | 8 | 91 | 19 |
|  | RV1+RV3 index |  |  |  |  |  |  |  | 38 | 2 | 91 | 19 |
| Amadis et al. 2024 [41] | Earliest onset or peak/ nadir in lead V2 | Indonesia, China | Jan 2017 to Jul 2022 | 45.0±14.0 | 0.75 | 65 | 18 | 47 | 6 | 12 | 34 | 13 |
|  | V2 R-wave duration index+R/S amplitude index |  |  |  |  |  |  |  | 11 | 7 | 35 | 12 |
|  | S-R difference in V1-V2 |  |  |  |  |  |  |  | 11 | 7 | 29 | 18 |
|  | RWDI |  |  |  |  |  |  |  | 0 | 18 | 47 | 0 |
|  | V2 transition ratio |  |  |  |  |  |  |  | 16 | 2 | 33 | 14 |
|  | TZI |  |  |  |  |  |  |  | 10 | 8 | 41 | 6 |
|  | V2S/V3R |  |  |  |  |  |  |  | 7 | 11 | 41 | 6 |
|  | V2QRS_i40_ |  |  |  |  |  |  |  | 5 | 13 | 41 | 6 |
|  | Combined index |  |  |  |  |  |  |  | 10 | 8 | 42 | 5 |
|  | Multistep algorithm |  |  |  |  |  |  |  | 12 | 6 | 44 | 3 |
| Zhang et al.2023 [34] | V2QRS_i40_ | China | Jun 2020 to Oct 2022 |  | 0.52 | 100 | 39 | 61 | 23 | 16 | 46 | 15 |
|  | R-wave duration index |  |  |  |  |  |  |  | 33 | 6 | 49 | 12 |
|  | R/S amplitude index |  |  |  |  |  |  |  | 38 | 1 | 33 | 28 |
|  | TZI |  |  |  |  |  |  |  | 29 | 10 | 49 | 12 |
|  | V2S/V3R |  |  |  |  |  |  |  | 37 | 2 | 54 | 7 |
|  | S-R difference in V1-V2 |  |  |  |  |  |  |  | 34 | 5 | 51 | 10 |
|  | ISA |  |  |  |  |  |  |  | 36 | 3 | 56 | 5 |
| Qiu et al.2023 [35] | V2S/V3R | China | Feb 2017 to May 2019 | 46.9±14.7 | 0.67 | 147 | 21 | 126 | 17 | 4 | 113 | 13 |
|  | TZI |  |  |  |  |  |  |  | 17 | 4 | 112 | 14 |
|  | Combined index |  |  |  |  |  |  |  | 5 | 16 | 109 | 17 |
|  | S-R difference in V1-V2 |  |  |  |  |  |  |  | 6 | 15 | 103 | 23 |
|  | R amplitude in lead I |  |  |  |  |  |  |  | 2 | 19 | 125 | 1 |
|  | ISA |  |  |  |  |  |  |  | 21 | 0 | 1 | 125 |
|  | V1 R/S amplitude index |  |  |  |  |  |  |  | 5 | 16 | 104 | 22 |
| Bourquin et al.2023 [24] | Clinical score | Switzerland | Jan 2013 to Jun 2019 | 52.0±15.0 | 0.55 | 187 | 64 | 123 | 47 | 17 | 82 | 41 |
|  | R/S transition |  |  |  |  |  |  |  | 56 | 8 | 102 | 21 |
| Gabriels et al.2021 [3] | V2S/V3R | USA | Jun 2014 to Jun 2018 | 52.0±16.0 | 0.53 | 109 | 38 | 71 | 24 | 14 | 63 | 8 |
|  | V2 transition Ratio |  |  |  |  |  |  |  | 34 | 4 | 37 | 34 |
|  | TZI |  |  |  |  |  |  |  | 25 | 13 | 36 | 35 |
|  | R-wave duration index |  |  |  |  |  |  |  | 11 | 27 | 67 | 4 |
|  | R/S amplitude index |  |  |  |  |  |  |  | 21 | 17 | 64 | 7 |
| Xia et al.2020 [6] | V2QRS_i40_ | China | Jan 2016 to Sep 2019 |  | 0.61 | 382 | 100 | 282 | 86 | 14 | 267 | 15 |
|  | V2S/V3R |  |  |  |  |  |  |  | 86 | 14 | 233 | 49 |
|  | V2 transition ratio |  |  |  |  |  |  |  | 90 | 10 | 163 | 119 |
|  | TZI |  |  |  |  |  |  |  | 64 | 36 | 220 | 62 |
| Nikoo et al. 2019 [33] | ISA | Iran | Jan 2010 to Jan 2015 | 44.8±13.9 | 0.47 | 60 | 23 | 37 | 18 | 5 | 35 | 2 |
|  | V2S/V3R |  |  |  |  |  |  |  | 19 | 4 | 34 | 3 |
|  | R-wave duration index |  |  |  |  |  |  |  | 16 | 7 | 31 | 6 |
| He et al.2018 [31] | Combined index | China | Aug 2008 to Dec 2015 | 41.3±13.1 | 0.68 | 488 | 49 | 439 | 40 | 9 | 376 | 63 |
|  | R-wave duration index |  |  |  |  |  |  |  | 29 | 20 | 387 | 52 |
|  | R/S amplitude index |  |  |  |  |  |  |  | 37 | 12 | 326 | 113 |
|  | TZI |  |  |  |  |  |  |  | 33 | 16 | 388 | 51 |
|  | Corrected TZI |  |  |  |  |  |  |  | 40 | 9 | 264 | 175 |
|  | V2S/V3R |  |  |  |  |  |  |  | 32 | 17 | 381 | 58 |
|  | V2 transition ratio |  |  |  |  |  |  |  | 39 | 10 | 300 | 139 |
| Celikyurt et al.2018 [25] | R/S amplitude index | Turkey | 2016-2018 |  |  | 32 | 9 | 23 | 7 | 2 | 13 | 10 |
|  | R-wave duration index |  |  |  |  |  |  |  | 6 | 3 | 19 | 4 |
|  | V2 transition ratio |  |  |  |  |  |  |  | 6 | 3 | 11 | 12 |
|  | V2S/V3R |  |  |  |  |  |  |  | 5 | 4 | 12 | 11 |
| Xie et al. 2018 [28] | R amplitude in lead I | USA | Jan 2009 to Nov 2015 |  | 0.65 | 70 | 16 | 54 | 12 | 4 | 53 | 1 |
|  | TZI |  |  |  |  |  |  |  | 8 | 8 | 41 | 13 |
|  | V2S/V3R |  |  |  |  |  |  |  | 11 | 5 | 48 | 6 |
| Ludwik et al. 2017 [29] | TZI | Poland | Sep 2009 to Nov 2016 | 45.0±16.7 | 0.66 | 202 | 72 | 130 | 67 | 5 | 110 | 20 |
|  | V2S/V3R |  |  |  |  |  |  |  | 65 | 7 | 119 | 11 |
|  | TZI+V2S/V3R |  |  |  |  |  |  |  | 63 | 9 | 127 | 3 |
| Lin et al. 2017 [26] | TZI | China | Jan 2008 to Mar 2016 | 50.4±15.6 | 0.55 | 49 | 23 | 26 | 22 | 1 | 25 | 1 |
|  | R/S transition |  |  |  |  |  |  |  | 20 | 3 | 16 | 10 |
|  | V2 R wave duration index+R/S amplitude index |  |  |  |  |  |  |  | 18 | 5 | 23 | 3 |
| Jiao et al. 2016 [30] | TZI | China |  | 45.3±15.3 | 0.54 | 110 | 26 | 84 | 16 | 10 | 78 | 6 |
|  | V2 transition ratio |  |  |  |  |  |  |  | 24 | 2 | 61 | 23 |
|  | V2 R wave duration index+R/S amplitude index |  |  |  |  |  |  |  | 21 | 5 | 79 | 5 |
| Yoshida et al. 2014 [32] | V2S/V3R | USA |  |  | 0.6 | 207 | 53 | 154 | 47 | 6 | 145 | 9 |
|  | TZI |  |  |  |  |  |  |  | 44 | 9 | 143 | 11 |
|  | R/S amplitude index |  |  |  |  |  |  |  | 42 | 11 | 132 | 22 |
|  | R-wave duration index |  |  |  |  |  |  |  | 24 | 29 | 142 | 12 |
| Yoshida et al. 2011 [27] | TZI | Japan | Apr 2003 to Oct 2009 | 48.0±17.0 | 0.5 | 112 | 25 | 87 | 22 | 3 | 71 | 16 |
|  | R-wave duration index |  |  |  |  |  |  |  | 11 | 14 | 74 | 13 |
|  | R/S amplitude index |  |  |  |  |  |  |  | 17 | 8 | 69 | 18 |

FN: false negatives; FP: false positives; ISA: initial r wave surface area; LVOT: left ventricular outflow tract; RVOT: right ventricular outflow tract; RWDI: R-wave deflection interval; TN: true negatives; TP: true positives; TZI: transitional zone index; UK: United Kingdom; USA: United States of America

**Appendix 4. Definitions of 21 ECG algorithms**

| **ECG algorithms** | **Definitions** |
| --- | --- |
| 1. TZI | TZI was calculated as the difference between the TZ (the precordial lead where the R/S-waves amplitude ratio is 0.9-1.1.) scores of the sinus beat and the OTVA; TZ score is the same as lead number when the TZ is located in the precordial lead. When the TZ occurs between two precordial leads, the TZ score is defined as n+0.5-point (n being the same as the lead number of the previous adjacent lead);  TZI <0 indicates an LVOT origin |
| 1. V2S/V3R | S-wave amplitude in lead V2 divided by the R-wave amplitude in lead V3;  ≤1.5 predicts an LVOT origin |
| 1. TZI+V2S/V3R | TZI <0 and V2S/V3R≤1.5 predicts an LVOT origin |
| 1. V2 transition ratio | It is calculated by computing the V2R-wave and V2S-wave amplitudes during VA, (R/R+S)_VA_ divided by those in SR, (R/R+S)_SR_;  ≥0.6 predicts an LVOT origin |
| 1. S-R difference in V1 and V2 | (V1S + V2S) – (V1R + V2R) ≤1.625mV predicts an LVOT origin |
| 1. R/S amplitude index | It is defined as the greater value of the R/S-wave amplitude ratio in lead V1 or V2;  ≥0.5 predicts an LVOT origin |
| 1. R-wave duration index | It is calculated by dividing the longer R-wave duration in lead V1 or V2 by the QRS duration;  ≥0.5 predicts an LVOT origin |
| 1. Combined index | −1.15×(TZI)−0.494×(V2S/V3R);  ≥−0.76 predicts an LVOT origin |
| 1. V2 R-wave duration index + R/S amplitude index | Lead V2 R-wave duration index >0.5 and R/S amplitude index >0.3 predicts an LVOT origin |
| 1. ISA | The higher value calculated by the R wave duration multiplies by the R wave amplitude in leads V1 or V2;  ≥15 predicts an LVOT origin |
| 1. R amplitude in lead I | R-wave amplitude in the lead I ≥0.1mV predicts an LVOT origin |
| 1. Clinical score | The score consists of three clinical parameters (hypertension, male sex and age >50 years), each equally weighted 1 score;  Score of ≥2 predicts an LVOT origin |
| 1. Corrected TZI | Corrected TZ score = n + $\frac{1-\left( \frac{R}{S} \right)V_{n}}{(\frac{R}{S)V_{n+1}}- (\frac{R}{S)V_{n}}}$  V_n_ and V_n+1_ representing the adjacent leads  Corrected TZI is calculated as the difference between the Corrected TZ scores of the sinus beat and the OTVA;  Corrected TZI <0 indicates an LVOT origin |
| 1. Earliest onset or peak/nadir in lead V2 | The earliest onset with earlier initial peak/nadir of QRS complex in V2 suggests an RVOT origin |
| 1. Hybrid algorithm | The Hybrid algorithm first analyzed the R/S transition: an early transition (R/S in V1–V2) indicated an LVOT origin. For cases with R/S transition in lead V3, a secondary step utilized the clinical score based on age (>50 years, 1 point), male gender (1 point), and hypertension (1 point);  Scores of 2–3 indicated an LVOT origin |
| 1. R/S transition | It is defined as the precordial lead (lead V1-V6) where the R/S-wave amplitude ratio during OVTAs is 0.9-1.1;  ≤V3 predicts an LVOT origin |
| 1. RWDI | It is defined as the lead V3 R-wave deflection interval (the beginning of QRS to the  peak R-wave interval in lead V3) and lead V1 R-wave amplitude;  A lead V3 RWDI of >80ms; lead V1 R-wave amplitude >0.3 predicted an LVOT origin |
| 1. V2QRS_i40_ | The amplitude of the QRS complex within the initial 40ms in lead V2;  ≥0.52 mV predicts an LVOT origin |
| 1. Weighted hybrid score (WHS) | 1. Clinical Parameters (maximum of +3 points):   +1 for age > 50 years  +1 for male gender  +1 for the presence of hypertension   1. Electrocardiographic Parameters (range from -4 to +3 points):   +3 for an R/S transition in lead V1  +2 for an R/S transition in lead V2  +1 for an R/S transition in lead V3 with a V3 R-wave amplitude ≥ 1 mV  -1 for an R/S transition in lead V3 with a V3 R-wave amplitude < 1 mV  -2 for an R/S transition in lead V4  -3 for an R/S transition in lead V5  -4 for an R/S transition in lead V6;  An LVOT origin is suggested if the total WHS is ≥ 2 points |
| 1. RV1+RV3 index | Summed R-wave amplitude in leads V1 and V3;  >1.3mV predicts an LVOT origin |
| 1. Multistep algorithm | 1. If the V₂ transition ratio is < 0.6 mV, RVOT is diagnosed; 2. If the V₂ transition ratio is ≥0.6 mV, assess whether the V2QRS_i40_ is < 0.52 mV; 3. If so, evaluate whether the earliest onset of QRS or the peak occurs in lead V₂. If this condition is met, RVOT is diagnosed. |

ISA: initial r wave surface area; RWDI: R-wave deflection interval; TZI: transitional zone index

**Appendix 5:**


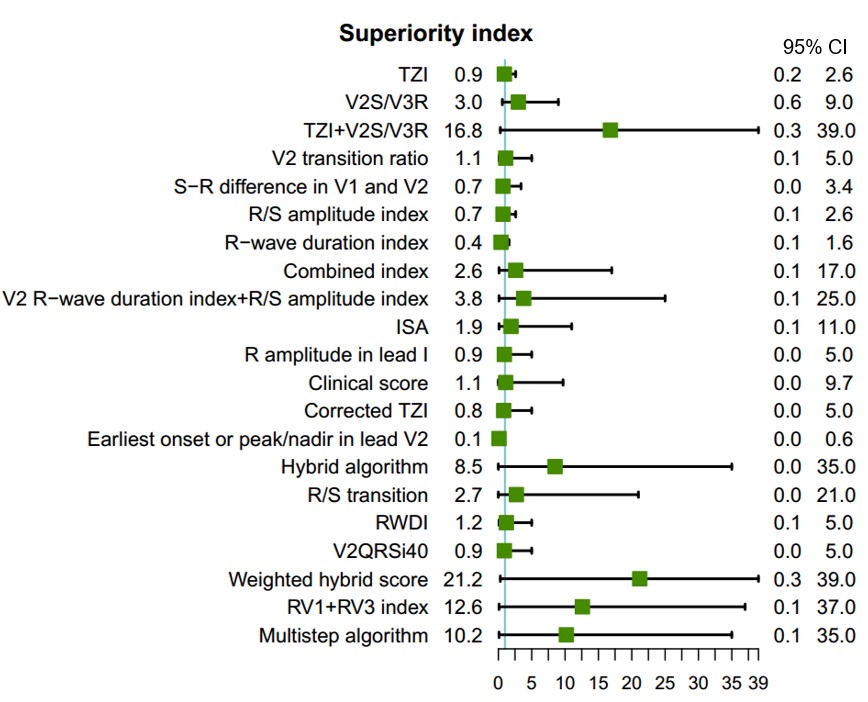


**Forest plot for superiority index of ECG algorithms**

ISA: initial r wave surface area; RWDI: R-wave deflection interval; TZI: transitional zone index

**Appendix 6: Relative sensitivity and specificity of various ECG algorithms compared to** **the** **‘Weighted hybrid score’ from the network meta-analysis**

| ECG algorithms | Relative sensitivity  (95% CI) | Relative specificity  (95% CI) |
| --- | --- | --- |
| 1. TZI | 0.90 (0.72, 1.40) | 0.89 (0.79, 1.16) |
| 2. V2S/V3R | 0.95 (0.75, 1.45) | 0.94 (0.83, 1.22) |
| 3. TZI+V2S/V3R | 0.93 (0.49, 1.49) | 1.04 (0.82, 1.37) |
| 4. V2 transition ratio | 1.06 (0.84, 1.63) | 0.72 (0.59, 0.96) |
| 5. S-R difference in V1 and V2 | 0.84 (0.54, 1.33) | 0.83 (0.63, 1.14) |
| 6. R/S amplitude index | 0.91 (0.69, 1.40) | 0.86 (0.73, 1.13) |
| 7. R-wave duration index | 0.75 (0.56, 1.14) | 0.92 (0.79, 1.24) |
| 8. Combined index | 0.88 (0.61, 1.43) | 0.95 (0.76, 1.27) |
| 9. V2 R-wave duration index + R/S amplitude index | 0.91 (0.60, 1.45) | 0.92 (0.70, 1.22) |
| 10. ISA | 1.11 (0.84, 1.74) | 0.64 (0.37, 0.94) |
| 11. R amplitude in lead I | 0.67 (0.38, 1.09) | 0.96 (0.76, 1.27) |
| 12. Clinical score | 0.84 (0.38, 1.45) | 0.68 (0.29, 1.08) |
| 13. Corrected TZI | 1.00 (0.69, 1.56) | 0.64 (0.35, 0.95) |
| 14. Earliest onset or peak/nadir in lead V2 | 0.47 (0.19, 0.88) | 0.81 (0.50, 1.17) |
| 15. Hybrid algorithm | 0.93 (0.49, 1.55) | 0.95 (0.62, 1.31) |
| 16. R/S transition | 1.01 (0.67, 1.67) | 0.78 (0.46, 1.12) |
| 17. RWDI | 0.34 (0.11, 0.71) | 1.08 (0.94, 1.43) |
| 18. V2QRS_i40_ | 0.73 (0.44, 1.22) | 0.93 (0.73, 1.25) |
| 19. Weighted hybrid score | 1.00 (1.00, 1.00) | 1.00 (1.00, 1.00) |
| 20. RV1+RV3 index | 1.13 (0.80, 1.76) | 0.84 (0.44, 1.23) |
| 21. Multistep algorithm | 0.92 (0.46, 1.54) | 0.99 (0.68, 1.32) |

ISA: initial r wave surface area; RWDI: R-wave deflection interval; TZI: transitional zone index

**Appendix 7:**


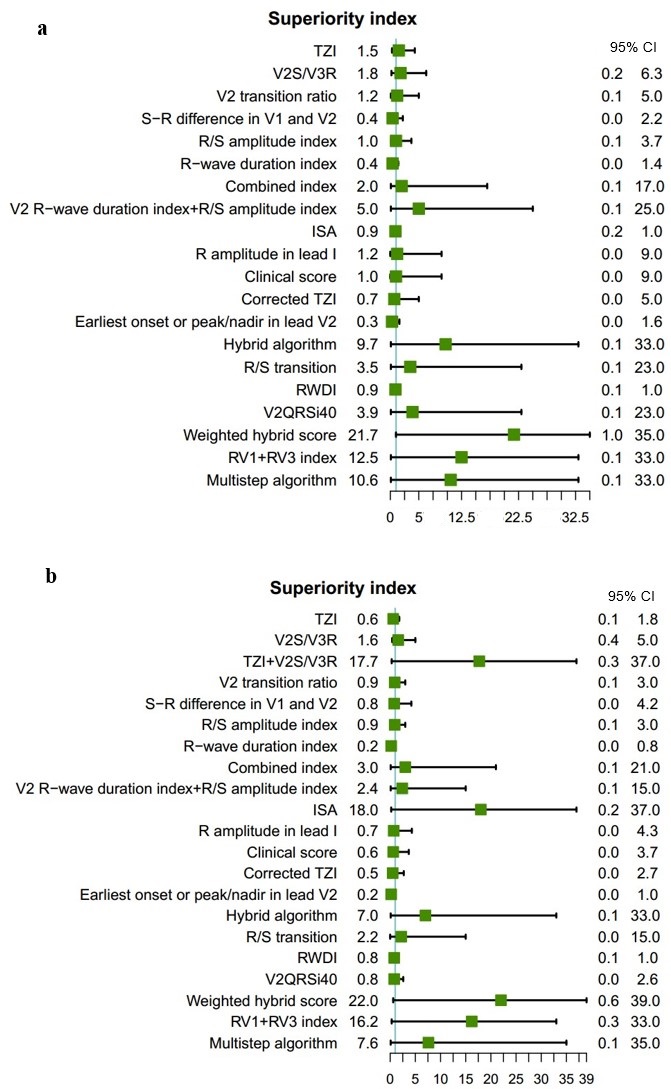


**Forest plots showing the superiority index of ECG algorithms in subgroup analyses**: (a) Excluding studies that included patients with OTVA exhibiting RBBB morphology. The ‘TZI+V2S/V3R’ algorithm was not assessed in this subgroup. (b) Excluding studies that defined RFCA success solely based on the acute suppression of OTVA, without considering long-term freedom from recurrence.

ISA: initial r wave surface area; RWDI: R-wave deflection interval; TZI: transitional zone index
